# Gastric Fundal Arteriovenous Malformation Presenting as Isolated Anemia and Dyspnea

**DOI:** 10.7759/cureus.93959

**Published:** 2025-10-06

**Authors:** Adit Railkar, Mithil Sheth, Kathryn Cooper

**Affiliations:** 1 Internal Medicine and Research, William Carey University College of Osteopathic Medicine, Hattiesburg, USA; 2 Emergency Medicine, William Carey University College of Osteopathic Medicine, Hattiesburg, USA; 3 Hospital Medicine, St. Tammany Parish Hospital, Covington, USA

**Keywords:** anemia, dieulafoy’s lesion, esophagogastroduodenoscopy, gastrointestinal bleed, shortness of breath

## Abstract

Arteriovenous malformations (AVMs) are rare, abnormal connections between arteries and veins. This aberrant circulation leads to rapid arterial blood flow into the venous system, resulting in inadequate tissue oxygenation and increased intravascular pressure, which can predispose to rupture. AVMs are typically located in the brain or spinal cord; however, this case highlights an atypical location in the gastric fundus. Known as a Dieulafoy’s lesion, this gastrointestinal (GI) AVM represents a rare, clinically elusive diagnosis in the setting of an unclear or unexplained GI bleed. A 62-year-old male patient with a history of five coronary artery bypass grafts (CABG), paroxysmal atrial fibrillation (PAF), heart failure with preserved ejection fraction (HFpEF), hypertriglyceridemia, and prior GI bleed presented to the emergency room with complaints of shortness of breath, worse with exertion, over the last three weeks, with associated pre-syncopal episodes and palpitations. Laboratory work and esophagogastroduodenoscopy (EGD) confirmed a 2 mm bleeding gastric fundal AVM. Similar cases of this lesion have been reported in the literature, though variations exist, as will be described later in the discussion. This case underscores the importance of considering uncommon GI sources of bleeding, such as Dieulafoy's lesion, in patients with unexplained anemia and cardiopulmonary symptoms.

## Introduction

Arteriovenous malformations (AVMs) are rare vascular anomalies characterized by direct connections between arteries and veins, bypassing the normal intervening capillary network [1]. The absence of an intervening capillary bed results in high-flow shunting of blood from arteries to veins. As a result, the lack of a capillary network prevents the normal pressure drop between arteries and veins, leading to elevated vascular pressure and an increased risk of AVM rupture. AVMs may also occur within the gastrointestinal (GI) tract, where they can serve as an occult source of bleeding and often present clinically as iron-deficiency anemia secondary to blood loss [2]. Gastric fundal AVMs are especially rare, representing roughly 1.5% of all GI AVMs [3]. Therefore, the presenting symptoms of the patient in this case were suspicious for a potential GI cause of anemia, although they did not immediately suggest an AVM as the underlying cause. Another rare yet potential source of a GI bleed is a Dieulafoy’s lesion, a markedly dilated or tortuous submucosal artery that becomes exposed through a mucosal defect, typically in the absence of adjacent ulceration [4]. The symptoms reported were shortness of breath on exertion, pre-syncopal episodes, and palpitations. Upper GI bleeds typically present with abdominal pain, hematemesis, or melena, which this patient denied [4].

## Case presentation

The patient was a 62-year-old male with a history of paroxysmal atrial fibrillation (PAF), five-vessel coronary artery bypass grafts (CABG), heart failure with preserved ejection fraction (HFpEF), and prior GI bleed. He presented to the emergency department (ED) with complaints of shortness of breath, worse with exertion, over the last several weeks. He reported associated dizziness, palpitations, and pre-syncopal episodes. He denied abdominal pain, nausea, vomiting, melena, hematochezia, hemoptysis, hematemesis, or hematuria. The patient was on rivaroxaban and aspirin, despite a history of GI bleed in 2021, due to a high-risk cardiac history. There was no active bleeding seen on endoscopy at that time in 2021, and the patient was discharged on 40 mg daily of pantoprazole with no further intervention needed.

**Figure 1:**
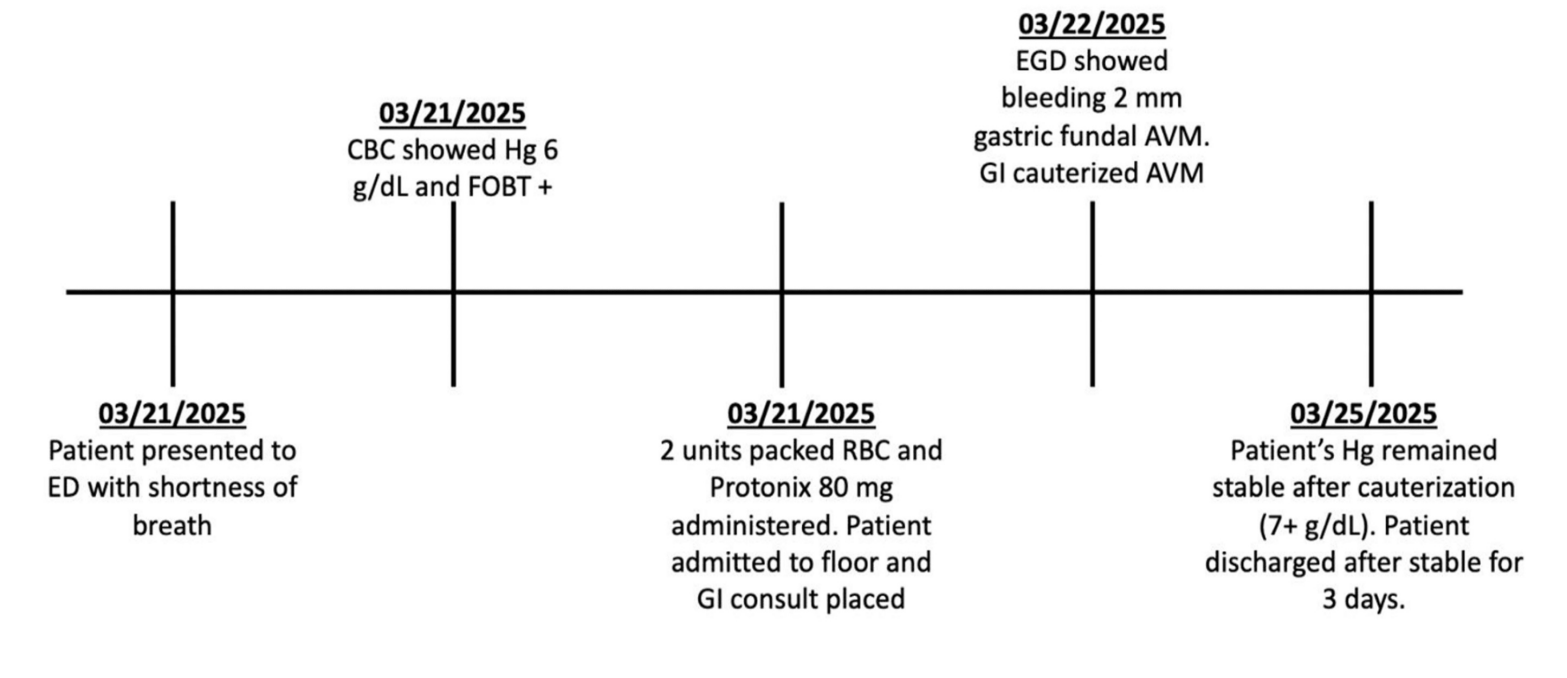
Chronological summary of the patient’s clinical course. ED: emergency department, CBC: complete blood count, Hg: hemoglobin, FOBT+: fecal occult blood test positive, RBC: red blood cell, GI: gastrointestinal, EGD: esophagogastroduodenoscopy, AVM: arteriovenous malformation

The patient’s vital signs on presentation were blood pressure 87/55 mmHg, temperature 98.3°F (36.8°C), respiratory rate 18, pulse 71, and SpO2 95%. The patient was noted to be pale on examination but exhibited no signs of acute distress. He had no abdominal tenderness or masses, and bowel sounds were normal. Cardiac examination revealed tachycardia but no murmurs or rhythm abnormalities. Lungs were clear to auscultation bilaterally. He had guaiac-positive stool, but no active bleeding from the rectum was noted. His full hospital course is summarized in Figure 1.

**Figure 2:**
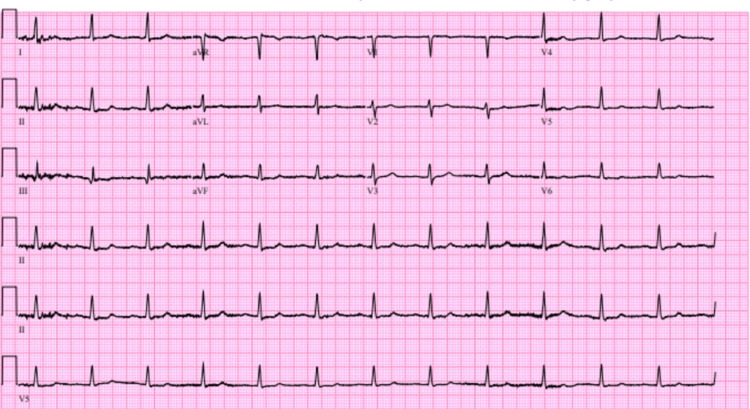
Patient’s electrocardiogram.

He was admitted for anemia in the setting of an unclear source of potential vascular or GI blood loss. Labs were remarkable for a hemoglobin of 6 g/dL and a positive fecal occult blood test (FOBT). In addition, the patient’s iron level was low at 33 mcg/dL, along with an elevated total iron binding capacity (TIBC) of 548 mcg/dL, with a ferritin level of 19 ng/mL and transferrin saturation of 8%. These significant lab values are summarized in Table 1, with corresponding reference ranges. In the setting of the patient’s clinical presentation of dyspnea on exertion, tachycardia, and pre-syncopal episodes, these lab values suggested a GI source of blood loss. The electrocardiogram (EKG) showed a junctional rhythm with low-voltage QRS complexes, as shown in Figure 2. The patient was on rivaroxaban for his history of atrial fibrillation; however, this was held for a week in an attempt to limit further bleeding.

**Table 1:** Significant laboratory results and corresponding reference ranges. TIBC: total iron binding capacity

Test	Value	Unit	Reference
Hemoglobin	6	g/dL	13.5-17.5
Serum Iron	33	mcg/dL	65-175
TIBC	548	mcg/dL	250-450
Ferritin	19	ng/mL	30-400
Transferrin Saturation	8	%	20-50

Imaging via abdominal ultrasound revealed hepatomegaly with diffuse hepatic steatosis, likely secondary to a history of hypertriglyceridemia, without evidence of obvious GI bleeding. Subsequently, an esophagogastroduodenoscopy (EGD) was performed due to suspicion of upper GI bleed, which showed a 2 mm bleeding gastric fundal AVM, as shown in Figure 2. Colonoscopy was considered but not performed due to the nature of the blood seen on the rectal exam performed in the ED (bright red). In the setting of acute hemodynamic instability with hypotension, tachypnea, and absence of chronic hematochezia, it was determined that the bright red blood observed on rectal exam in the ED was likely due to reduced transit time of the blood from the gastric fundal AVM.

**Figure 3:**
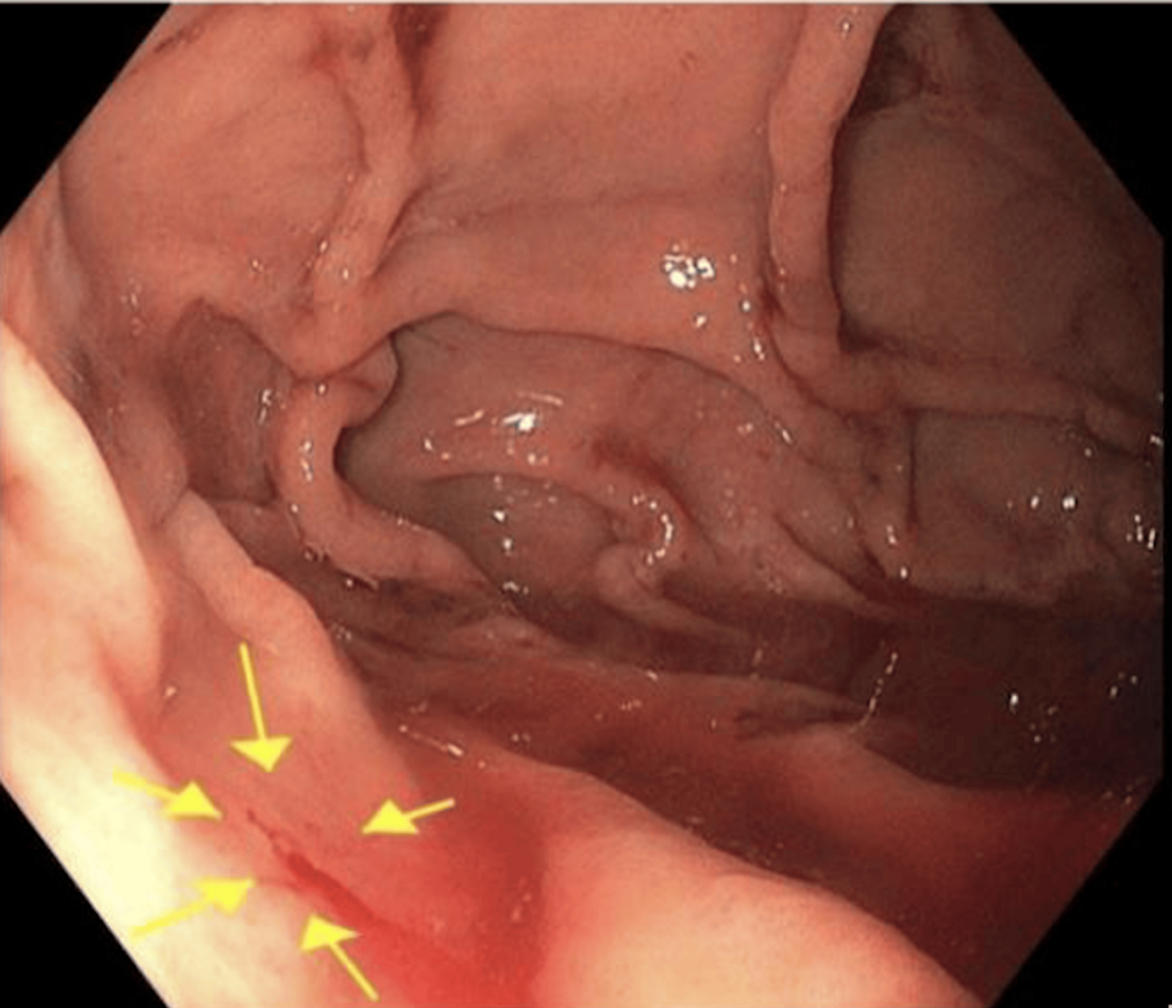
EGD depicting a bleeding 2 mm gastric fundal AVM. EGD, esophagogastroduodenoscopy; AVM, arteriovenous malformation

Initially, he was treated with two units of packed red blood cells (pRBC) and 80 mg intravenous (IV) pantoprazole in the ED, in the setting of anemia with a hemoglobin level under 7 g/dL. After the AVM was identified on EGD, it was cauterized with argon gas on hospital day two. Following the EGD, as shown in Figure 3, the patient developed hypotension. In response, cardiology discontinued spironolactone 25 mg and torsemide 40 mg, both administered orally once daily, and reduced his carvedilol dose from 12.5 mg to 6.25 mg twice daily. His blood pressure remained stable following these medication adjustments. The patient did not develop any further post-operative complications. Following successful cauterization of the bleeding gastric fundal AVM, the patient reported significant symptomatic improvement, specifically noting resolution of his presyncopal episodes, dizziness, and shortness of breath. Given the patient’s symptomatic improvement and return to hemodynamic stability after cauterization, the nature of the initial bright red blood on rectal exam in the ED was concluded to be secondary to massive hemorrhage from the gastric fundal AVM. He expressed relief and satisfaction with the outcome and demonstrated a clear understanding of the importance of follow-up care with his specialists to manage his ongoing chronic conditions.

The patient was adherent to his discharge plan and has remained stable as of the follow-up visit in April 2025. He restarted his anticoagulation medication for his PAF about 72 hours after cauterization and was given oral ferrous sulfate tablets to take at home for iron deficiency anemia, with which he has been compliant.

## Discussion

This clinical case demonstrates a rare vascular phenomenon: gastric fundal AVM. GI AVMs are rare, with 80% involving the small or large intestine [2]. It has been reported that only roughly 1.5% of intestinal AVMs are gastric in origin [3]. Furthermore, this case highlights an atypical cause of upper GI bleeding. The typical causes of upper GI bleeds are gastric and duodenal ulcers (15-29% and 14-16%, respectively), as well as varices (5-33%), with AVMs of the stomach making up less than 5% of bleeds [5]. Of the uncommon causes of GI bleeds, Dieulafoy’s lesions are described in the literature to account for approximately 1-2% of these [6]. This lesion is defined as an abnormally enlarged submucosal artery that becomes exposed through a mucosal defect and erodes the surrounding tissue, resulting in significant arterial hemorrhage [4]. To identify this lesion, an EGD is typically required to visualize the tortuous artery and to provide an intervention to make the vessel hemostatic [4]. The lesion identified in this patient was similar to a Dieulafoy's lesion due to its aberrant, enlarged appearance and permeation through the mucosa without the presence of an ulcer. However, this patient’s vascular malformation was not just a tortuous, exposed artery, but rather an AVM. Therefore, it represented a unique form of Dieulafoy's lesion as an AVM. A similar classification of an underlying AVM as a Dieulafoy's lesion was reported in a 2017 case involving a 28-year-old male [3]. In that case, the patient presented with severe epigastric pain and melena. The underlying cause was determined to be an AVM presenting as a Dieulafoy’s lesion during EGD. Although the underlying cause was similar, the patient in this study presented with dyspnea and pre-syncopal episodes. The GI cause in this patient was first identified from a positive FOBT in the absence of overt GI symptoms.

In another previously published case, a 34-year-old male presented with fatigue, melena, and hematemesis for four days before admission [2]. This patient shared several clinical similarities with the current case, most notably a hemoglobin level below 7 g/dL and the absence of abdominal pain. Upper endoscopy revealed a submucosal lesion in the gastric fundus with an overlying ulcer; however, no Dieulafoy’s lesion was identified, in contrast to the patient in this study. Other key differences included the presence of hematemesis and melena, unlike the patient in this study.

## Conclusions

This case represents a unique underlying cause of a common ED problem: GI hemorrhage. Within the causes of GI hemorrhage, this patient had a unique anatomical pathology as a Dieulafoy’s lesion presenting as an AVM in the gastric fundus. Furthermore, the clinical presentation did not immediately suggest a GI root cause because the patient presented with dyspnea, palpitations, and pre-syncopal episodes. The potential differential diagnoses were broadened after the identification of a positive FOBT in the setting of anemia, with his hemoglobin below 7 g/dL. Therefore, this case highlights the importance of maintaining a broad differential when evaluating patients with non-specific cardiopulmonary symptoms and laboratory findings suggestive of a GI source.
